# Fibroblast Growth Factor 2 Modulates Hippocampal Microglia Activation in a Neuroinflammation Induced Model of Depression

**DOI:** 10.3389/fncel.2018.00255

**Published:** 2018-08-08

**Authors:** Ming-ming Tang, Wen-juan Lin, Yu-qin Pan, Ying-cong Li

**Affiliations:** ^1^CAS Key Laboratory of Mental Health, Institute of Psychology, Beijing, China; ^2^Department of Psychology, University of Chinese Academy of Sciences, Beijing, China; ^3^Institute of Zoology, Chinese Academy of Sciences, Beijing, China

**Keywords:** fibroblast growth factor 2, microglia, depressive-like behavior, neuroinflammation, cytokines, CX3CL1, hippocampus

## Abstract

Recent studies indicate that disturbed structure and function of microglia can cause depression and associated neurogenesis impairments. Our previous work has demonstrated that exogenous fibroblast growth factor 2 (FGF2) reverses the depressive-like behaviors and the impaired hippocampal neurogenesis in a neuroinflammatory model of depression. However, whether and how the antidepressant effects of FGF2 involve the modulation of microglia activation has not been elucidated. In this study, to examine the effects of FGF2 on microglia activation, exogenous FGF2 was supplemented to the lateral ventricle of rats during the neuroinflammatory state induced by central lipopolysaccharides (LPS) administrations. It was found that FGF2 infusions reversed the LPS-induced depressive-like behaviors and inhibited the hippocampal microglia activation. In LPS-treated rats, FGF2 decreased the level of pro-inflammatory cytokines including interlukin-1β (IL-1β), IL-6 and tumor necrosis factor (TNF)-α, increased the level of IL-10, the anti-inflammatory cytokine and reversed the decreased expression of CX3CL1, a chemokine mainly expressed by neurons and keeping microglia in surveillance. Further, we examined the effects of inhibited FGF2 signaling by administration of SU5402, an FGFR inhibitor. It was found that SU5402 itself evoked depressive-like behaviors, induced microglia activation, increased production of pro-inflammatory cytokines including IL-1β, IL-6 and TNF-α, and decreased the expression of CX3CL1. Two lines of results that FGF2 signaling and FGFR inhibitor can effectively but oppositely modulate the regulation of microglia and the generation of depressive-like behavior, suggesting that microglia-regulated mechanisms may underlie the antidepressant role of FGF2. The present data provide novel insights into the understanding of mechanism of neuroinflammation-associated depression and may serve as a novel mechanism-based target for the treatment of inflammation-related depression.

## Introduction

Neuroinflammation, the inflammation of the central nervous system (CNS), is considered as a fundamental process of many pathologic conditions in the brain, such as Alzheimer’s disease and major depressive disorder (Hurley and Tizabi, 2013; Morales et al., 2014). The neuroinflammatory response is primarily mediated via glia cells, particularly microglia which are the resident innate immune cells in the CNS. In the healthy CNS, microglia have highly ramified morphology with thin processes, which dynamically monitor the neural cell microenvironment for surveillance in efforts to maintain homeostasis (Nimmerjahn et al., 2005). In response to an infection or injury, microglia transform into a reactive inflammatory phenotype, which is also known as the classic activation or M1 phenotype that is characterized by increased proliferation, morphologic changes, and the release of inflammatory molecules such as cytokines, chemokines and reactive oxygen species (Kettenmann et al., 2011). Though the M1 phenotype intends to protect and repair the CNS from being damaged, it also can be cytotoxic and harmful to the neural microenvironment, even triggers neurodegenerative and neuropsychiatric diseases if excessive and prolonged neuroinflammation occurs (Berk et al., 2011; Czeh et al., 2011; Cherry et al., 2014).

Many researches indicate an association between microglia and the pathology of depression and suggest antidepressant properties of microglia modulation. Using different PET ligands, increased activated microglia were found in the prefrontal cortex (PFC) and hippocampus of depressive and bipolar disorder patients (Haarman et al., 2014; Setiawan et al., 2015). Chronic stress, which is causally related to the development of depression, could alter the number, morphology and functioning of microglia in hippocampus and PFC (Tynan et al., 2010; Hinwood et al., 2012; Pan et al., 2014; de Pablos et al., 2014). Antidepressants can decrease the production of pro-inflammatory cytokines and suppress microglia activation (Lim et al., 2009; Kreisel et al., 2014). Further, the microglial inhibitory drug minocycline was found to prevent the development of depressive-like symptoms in lipopolysaccharides (LPS) model of depression, and to ameliorate neurogenic defects and depressive-like behaviors in interferon-α model of depression (Henry et al., 2008; Zheng et al., 2015).

The healthy morphology and function of microglia could be impaired by intense inflammatory activation or chronic stress exposure, as well as could be controlled by signals delivered from nearby neurons. Such signals keep microglia in their surveillance phenotype indicating normal function (Biber et al., 2007; Gemma and Bachstetter, 2013). One of such signals is the fractalkine (CX3CL1), which is a chemokine constitutively expressed by neurons and has been considered as a novel neuroimmune regulatory factor (Limatola and Ransohoff, 2014). Depending on the disorder or the course of brain injury, CX3CL1 contributes to augment microglia pro-inflammatory response or maintain microglia in quiescence (Liu et al., 2015; Zanier et al., 2016; Dorfman et al., 2017). It has been suggested that CX3CL1 is involved in neuroprotection by sending alert signals to microglia and maintain microglia in their resting phenotype when the inflammation is overreacting (Ransohoff et al., 2007; Corona et al., 2010; Wynne et al., 2010). In the late stage of brain injury, mice with CX3CR1 deletion show chronic deterioration of injury outcome, possibly due to the lack of CX3CL1/CX3CR1 signal that reduces the microglial overactivation (Zanier et al., 2016).

Previously we have reported that exogenous fibroblast growth factor 2 (FGF2) supplement reverses the depressive-like behaviors and the inhibited hippocampal neurogenesis induced by neuroinflammation (Tang et al., 2017). FGF2 is a multi-functional growth factor and induces pleiotropic effects which include potent neurogenic effects and important roles in the function of the CNS (Woodbury and Ikezu, 2014). Though some studies suggest that FGF2 has potent effect not only on neurons but on glia (Downer et al., 2010; Cox et al., 2013), whether and how FGF2 affect microglia in neuroinflammation-associated depression remains elucidative. We hypothesize that the antidepressant effects of FGF2 and the neuroinflammation-induced depression may involve the modulation of microglia changed by FGF2 and FGFR inhibitor.

To examine whether FGF2 signaling affects microglia activation, in the present study, we gave five consecutive FGF2 central infusions to rats which were under neuroinflammatory status induced by triple central LPS administration. The depressive-like behaviors were measured and the changes of microglia activation in hippocampus were assessed. The ionized calcium-binding adaptor molecule-1 (Iba1) was used as microglia identification marker to measure the quantity and morphology of microglia. The production of pro-inflammatory and anti-inflammatory cytokines were analyzed to determine the changes of neural microenvironment induced by microglia activation. We also examined the expression of CX3CL1 protein, the neuro-immune regulatory chemokine. Further, to examine the effects of blocking FGF2 signaling, we gave infusions of SU5402, an inhibitor of FGFR activity, to lateral ventricle and accessed the behavioral effects, the microglia activation, production of cytokines, and expression of CX3CL1 protein in hippocampus.

## Materials and Methods

### Animals

Male Sprague-Dawley rats weighing 220–240 g were obtained from Vital River Laboratories (Beijing, China), and were individually housed in standard stainless steel cages with free access to food and water. The animal room is temperature and humidity controlled (22 ± 1°C, 40–60%) with a 12:12 dark/light cycle (lights on at 07:00, off at 19:00). To minimize the stress responses to the experimental manipulation, all rats were administered 5 min of daily handling for 1 week prior to experimental use. The experimental procedures were approved by the Institutional Review Board of the Institute of Psychology, Chinese Academy of Sciences and were consistent with the National Institutes of Health Guide for Care and Use of Laboratory Animals.

### Surgical Procedures

After acclimation, rats were anesthetized with pentobarbital sodium (Merck, Darmstadt, Germany; 1%, 35 mg/Kg; intraperitoneal injection). The top of the animal’s head was shaved and fixed in a stereotaxic instrument (Stoelting, Wood Dale, IL, USA) with the incisor bar set at 3.3 mm below the interaural line. Body temperature was maintained at 37°C using a rectal thermometer and a feedback-controlled heating pad (RWD, Shenzhen, China). Guide cannula was implanted into the lateral ventricle (AP: −0.9 mm from the Bregma; LM: 1.1 mm to the sagittal suture; DV: 3.2 mm in depth relative to the skull). A 1 mm diameter hole was drilled and the cannula with outer diameter 0.64 mm and inner diameter 0.45 mm (RWD Life Science Co., Ltd, Shenzhen, China) was unilaterally implanted above the lateral ventricle. Acrylic resin and two stainless steel screws were used to fix the guide cannula to the skull. A stylet with the same length as the guide cannula was inserted to prevent obstruction. After surgery, rats were returned to their home cages to recover for 10 days.

### Drug Infusion

LPS (derived from *E. coli* serotype 0111: B4, No: L-2880, Sigma, St Louis, MO, USA) was used as the pro-inflammatory cytokine-inducer and was infused intracerebroventricularly (i.c.v) at a dose of 100 ng/rat (100 ng/μl, diluted with sterile saline, flow rate 0.5 μl/min). The dosage was chosen based on our previous study demonstrating the efficacy to induce significantly depressive-like behaviors in rats (Tang et al., 2016, 2017). We gave infusions of LPS or saline to rats every other day. Recombinant human FGF2 (R&D Systems, Minneapolis, MN, USA) was infused i.c.v at a dose of 200 ng/rat/day (200 ng/μl, diluted with sterile saline, flow rate 0.5 μl/min). This FGF2 dosage has previously been shown to improve the depressive-like behavior and facilitate hippocampus neurogenesis (Elsayed et al., 2012; Tang et al., 2017). We gave FGF2 or vehicle (saline) infusions to rats for five consecutive days. SU5402 (R&D Systems, USA), the inhibitor of FGFR activity, was dissolved with dimethyl sulfoxide (DMSO) to 10 μg/μl. The solution was diluted to 2 μg/μl or 5 μg/μl by saline before use, and then was infused i.c.v at a dose of 2 μg/rat/day or 5 μg/rat/day (flow rate 0.25 μl/min). This dosage was chosen by reference to previous studies (Mudò et al., 2007; Fujita-Hamabe et al., 2011; Elsayed et al., 2012). Rats were infused with SU5402 or vehicle (20% DMSO in saline) for five consecutive days.

### Experimental Design

#### Experiment 1. The Effects of LPS And FGF2 on Depressive-Like Behaviors and Microglia Activation in Hippocampus

**Figure 1:**
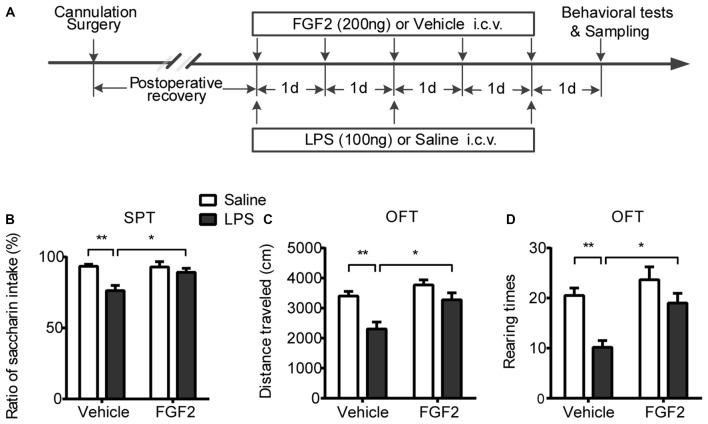
FGF2 reversed the depressive-like behaviors induced by LPS administration. **(A)** The timeline of Experiment 1. Group LPS + FGF2 received central FGF2 infusions once each day for five consecutive days during the central LPS administration. Three control groups of LPS + Vehicle, Saline + FGF2 and Saline + Vehicle received the corresponding treatments and were served to examine the effects of LPS, FGF2 and Vehicle infusions respectively. The behavioral tests and brain tissues sampling were carried out 24 h after infusions. **(B)** SPT. The decreased saccharin intake rate was reversed by FGF2 infusions in LPS-treated rats. **(C,D)** OFT. The decreased distance traveled and rearing times were reversed by FGF2 infusions in LPS-treated rats. Two-way ANOVA with Tukey’s multiple comparisons test, *n* = 6 per group. Data are presented as the means ± SEM. *p < 0.05, **p < 0.01. LPS, lipopolysaccharide; FGF2, fibroblast growth factor 2; i.c.v., intracerebroventricular; SPT, Saccharin preference test; OFT, Open field test.

After recovery from surgery, 40 rats were randomly divided into four groups (*n* = 10 per group). Group LPS + FGF2 received central FGF2 infusions once each day for five consecutive days during the central LPS administration as previously reported (Tang et al., 2017). Three control groups (LPS + Vehicle, Saline + FGF2 and Saline + Vehicle) received the corresponding treatments and were served to examine the effects of LPS, FGF2 and vehicle infusions respectively. Six rats from each group were subjected to behavioral tests 24 h after the central infusions. To eliminate the possible stress effects resulting from behavioral tests to interfere with molecular and cellular indicators, the other four rats per group without behavioral tests received cardiac perfusion 24 h after the central infusions for brain tissue sampling to examine microglia activation via immunohistochemistry method. The behavioral experiment and the brain sampling collections were conducted simultaneously. The experimental timeline was shown in Figure 1A.

#### Experiment 2. The Effects of LPS and FGF2 on Cytokines and CX3CL1 Expression in Hippocampus

After recovery from surgery, 24 rats were randomly divided into four groups (*n* = 6 per group): LPS + FGF2, LPS + Vehicle, Saline + FGF2 and Saline + Vehicle (as described in “Experiment 1. The Effects of LPS And FGF2 on Depressive-Like Behaviors and Microglia Activation in Hippocampus” section). All rats were sacrificed 24 h after the central infusions for fresh brain tissue sampling to determine cytokines and CX3CL1 expression via cytokines magnetic bead panel assay and western blot, respectively.

#### Experiment 3. The Effects of SU5402 on Depressive-Like Behaviors and Microglia Activation in Hippocampus

After recovery from surgery, 30 rats were randomly divided into three groups (*n* = 10 per group). Two groups received i.c.v. SU5402 infusions 2 μg or 5 μg respectively, for five consecutive days. DMSO was given as a control vehicle. Six rats from each group were subjected to behavioral tests 24 h after the central infusions. As the same procedures as described in Experiment 1, other four rats per group without behavioral tests received cardiac perfusion 24 h after the central infusions for brain tissue sampling to examine microglia activation via immunohistochemistry method. The experimental timeline was shown in Figure 4A.

**Figure 2:**
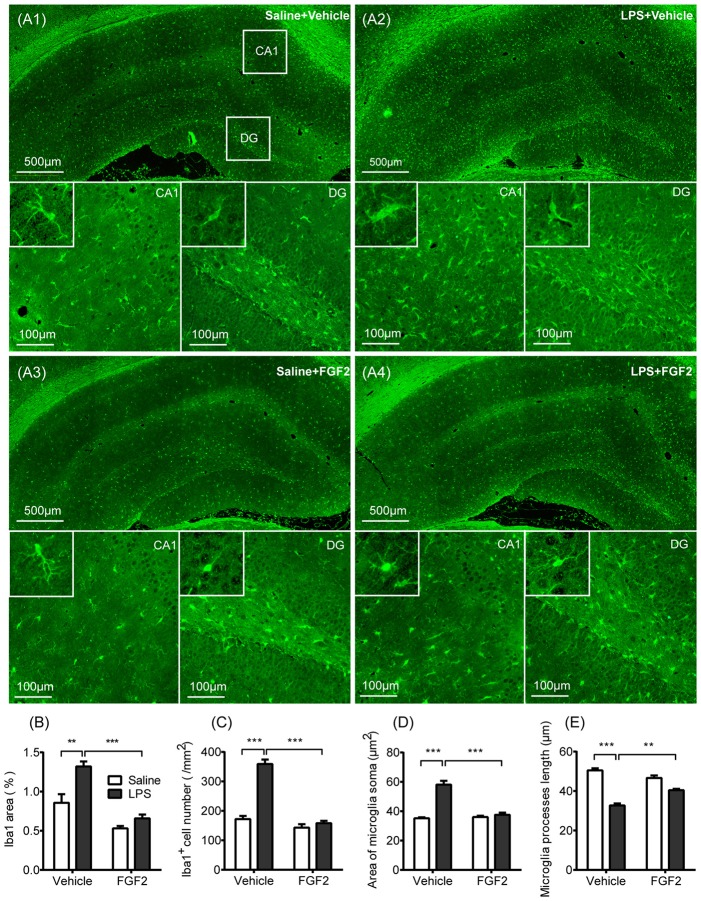
FGF2 decreased microglia activation in hippocampus induced by central LPS treatment. **(A1–A4)** Representative photographs of the ionized calcium-binding adaptor molecule-1 (Iba1) expression in hippocampus. In LPS + Vehicle group, there were increased expression of microglia identification marker Iba1 and more Iba1 positive cells in CA1 and DG with the swollen soma and retracted processes. In LPS + FGF2 group, the number and morphology of Iba1 positive cells were closer to the control group. **(B)** The comparison of the percentage of Iba1 positive area. The increased Iba1 area was reversed by FGF2 in LPS-treated rats. **(C)** The comparison of the density of Iba1 positive cells. The increased Iba1 cell density was reversed by FGF2 in LPS-treated rats. **(D)** The comparison of the microglial soma area. The increased soma area was reversed by FGF2 in LPS-treated rats. **(E)** The comparison of microglia processes length. The decreased processes length was reversed by FGF2 in LPS-treated rats. Two-way ANOVA with Tukey’s multiple comparisons test, *n* = 4 per group. Data are presented as the means ± SEM. **p < 0.01, ***p < 0.001. LPS, lipopolysaccharide; FGF2, fibroblast growth factor 2; DG, dentate gyrus.

**Figure 3:**
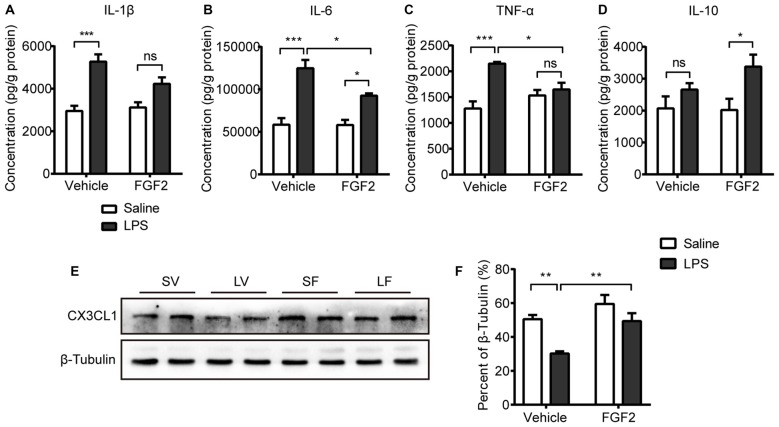
Impact of LPS and FGF2 on cytokines and CX3CL1 expression in hippocampus. **(A)** The level of interlukin-1β (IL-1β) was increased by LPS treatment. FGF2 prevented the IL-1β upregulation after LPS treatment. **(B)** The level of IL-6 was increased by LPS treatment. FGF2 alleviated the IL-6 upregulation after LPS treatment. **(C)** The level of tumor necrosis factor-α (TNF-α) was increased by LPS treatment. FGF2 reversed the increased TNF-α level induced by LPS treatment. **(D)** FGF2 increased the level of IL-10 in the LPS treated rats. **(E)** Representative western blots of CX3CL1 and corresponding normalized control β-Tubulin in hippocampus. **(F)** The expression of CX3CL1 was decreased by LPS treatment and was reversed by FGF2 infusions. Two-way ANOVA with Tukey’s multiple comparisons test, *n* = 5 or 6 per group. Data are presented as the means ± SEM. *p < 0.05, **p < 0.01, ***p < 0.001, ns non-significant. LPS, lipopolysaccharide; FGF2, fibroblast growth factor 2; SV, Saline + Vehicle; LV, LPS + Vehicle; SF, Saline + FGF2; LF, LPS + FGF2.

**Figure 4:**
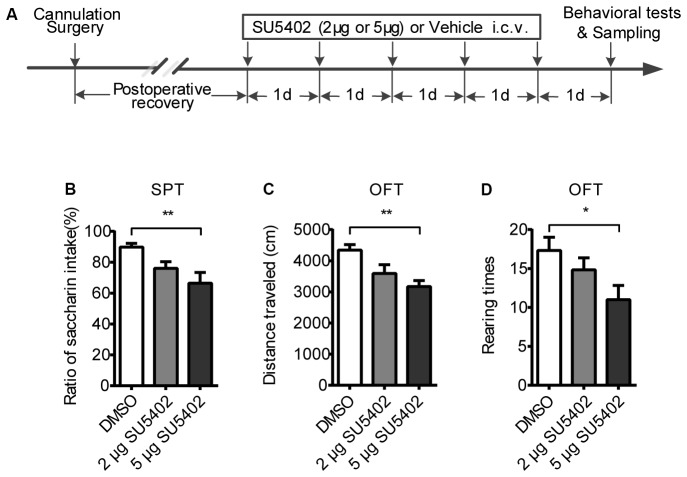
The behavioral effects of FGFR inhibitor (SU5402). **(A)** The timeline of Experiment 2. Two groups respectively received 2 μg or 5 μg doses of central SU5402 infusions for five consecutive days. Dimethyl sulfoxide (DMSO) was used as Vehicle in control group. **(B)** Saccharin preference test. A 5 μg dose of SU5402 infusions induced significant decreased ratio of saccharin intake. **(C,D)** OFT. A 5 μg dose of SU5402 infusions decreased the distance traveled and rearing times. One-way ANOVA with Dunnett’s multiple comparisons test, *n* = 6 per group. Data are presented as the means ± SEM. *p < 0.05, **p < 0.01. i.c.v., intracerebroventricular; SPT, Saccharin preference test; OFT, Open field test.

#### Experiment 4. The Effects of SU5402 on Cytokines and CX3CL1 Expression in Hippocampus

After recovery from surgery, 18 rats were randomly divided into three groups (*n* = 6 per group): DMSO group, 2 μg dose of SU5402group, and 5 μg dose of SU5402 group (as described in “Experiment 3. The Effects of SU5402 on Depressive-Like Behaviors and Microglia Activation In Hippocampus” section). All rats were sacrificed 24 h after the central infusions for fresh brain tissue sampling to determine cytokines and CX3CL1 expression via cytokines magnetic bead panel assay and western blot, respectively.

### Behavioral Tests

#### Saccharin Preference Test (SPT)

Decreased proportion of sweet solution intake is used to measure anhedonia, a core symptom of depression (Wann et al., 2010). The saccharin preference test was carried out as described in our previous studies (Tang et al., 2016, 2017). During the period of 3 days habituation, the rats were given a bottle of water and a second bottle of 0.5% saccharin solution for 4 h (8:00–12:00 a.m.) and deprived liquid supply at other times (20 h). On the test day, each rat was offered the two bottles (saccharin and water) for 1 h (8:00–9:00 a.m.) after a 20 h liquid deprivation. To avoid the positional preferences, positions of the bottles were exchanged every 30 min. After the tests, the bottles were weighed to calculate the liquid consumption. The saccharin preference was assessed as the ratio of saccharin intake to the total intake (saccharin solution plus water intake).

#### Open Field Test (OFT)

Reduction of locomotor activity is one of characteristic symptoms of major depression (Maes et al., 2012), and is linked to reductions in energy generation in the depressive episode of mood disorder (Caliyurt and Altiay, 2009). After the saccharin preference test, locomotor activity was measured for 5 min in an open field test (OFT) as described in our previous studies (Wang et al., 2011; Pan et al., 2013; Tang et al., 2016). The apparatus was a circular arena placed in a room with a dim illumination (40 W). The diameter of the area was 180 cm, and the height of the surrounding wall was 50 cm. We placed rat in the center of the field, recorded the distance traveled and the number of rearing using a computer-based system (Ethovision software, Noldus Information Technology, Wageningen, Netherlands). The apparatus was cleaned with a 30% ethanol solution after each test. The order of testing was balanced by group.

### Immunohistochemistry

The animals were deeply anesthetized via an intraperitoneal injection of 10% chloral hydrate solution and were cardiac perfused with 200 ml of phosphate-buffered saline (PBS), followed by 200 ml of 4% paraformaldehyde (PFA). The brains were then excised and post-fixed in 4% PFA at 4°C overnight. The brains were embedded in paraffin and cut into 5 μm thick sections (Verheijden et al., 2015; Robillard et al., 2016; Michailidou et al., 2017). The sections were dewaxed in xylene and rehydrated via immersion in a series of graded alcohols. For antigen unmasking, the sections in 10 mM citrate buffer (pH 6.0) were heated in a microwave oven at 80–85°C for 15 min, then were cooled down for 1 h at room temperature (RT). Serum blocking was performed by 5% normal rabbit serum solution for 1 h at RT. For Iba1 staining, sections were incubated with goat anti-Iba1 antibody (1:100, ab5076, Abcam, Cambridge, UK) for 2 h at 37°C. The sections were subsequently washed in PBS and incubated with fluorescein isothiocyanate (FITC)-conjugated rabbit anti-goat IgG (1:100, Jackson, West Grove, PA, USA) for 1 h at RT. Counterstaining was performed with 4′,6-diamidino-2-phenylindole (DAPI).

For analyzing the immunostaining in hippocampus, 10 coronal brain sections were examined per animal, from Bregma −3.00 mm to Bregma −3.60 mm (plates 58–63; Paxinos and Watson, 2006). All images were taken to two-dimensional pictures using a fluorescence microscope (Leica Microsystems, Wetzlar, Germany). Four indexes of microglia activation were calculated referencing to previous studies (Kreisel et al., 2014; Zhang et al., 2017). The percentage of Iba1 positive area and the density of Iba1 positive cells were automatically counted at ×10 magnification using Image Pro-plus software (Media Cybernetics, Bethesda, MD, USA) in a defined area containing the hippocampus. The area of microglia soma and the microglia processes length were measured at ×20 magnification in five randomly selected cells/section using the Image Pro-plus software.

### Cytokines Magnetic Bead Panel Assay

The levels of cytokines (interlukin-1β (IL-1β), IL-6, TNF-α and IL-10) in hippocampus were determined by the MILLIPLEX MAG rat cytokine magnetic bead array panel (#RECYTMAG-65K-04, Millipore, Billerica, MA, USA). The tissue samples were homogenized in 0.01 Mol/L PBS (pH 7.4) containing a protease inhibitor cocktail (#535140–1MLCN, Millipore). The lysates were then centrifuged at 15,000× *g* for 20 min at 4°C. The protein concentrations of the lysates were determined using BCA assay kits (Thermo Fisher Scientific Inc., Waltham, MA, USA). The cytokines levels in the extracts from homogenized brains were examined used the magnetic bead panel assay as previously described (Wei et al., 2013; Chai et al., 2014). In brief, premixed magnetic beads conjugated to antibodies for all four analytes were mixed with equal volumes of brain lysates in 96-well plate in duplicate, and were incubated on a plate shaker overnight at 4°C. Magnetic beads were washed with 200 μl of wash buffer three times. Then detection antibodies were added to each well, and the plate was incubated at RT for 1 h. Streptavidin-Phycoerythrin was added to each well, and the mixtures were incubated on a plate shaker at RT for 30 min. The magnetic beads were resuspended in sheath fluid, and plates were read on FLEXMAP 3D™ system with xPONENT software. Median fluorescence intensity data were analyzed using a spline curve-fitting method for calculating cytokines concentrations in brain homogenates. The results were expressed as pg/g protein.

### Western Blot

The western blot was carried out as described in our previous studies (Tang et al., 2016, 2017). The brain tissue lysates acquired using the same method described in the above procedure were mixed with 5× loading buffer to prepare for specific concentrations of the sample solutions. The proteins were separated by sodium dodecyl sulfate polyacrylamide gel electrophoresis on 12% polyacrylamide gels and, were then moved to a 0.2 μm nitrocellulose filter (NC; Sigma-Aldrich, St. Louis, MO, USA) by electrophoretic transfer. Blocking buffer (5% non-fat dry milk powder in Tris-buffered saline containing 0.05% Tween-20, TBST) was used to incubate the NC overnight at 4°C. Then NC was washed for 10 min ×3 in TBST and was incubated with primary antibody, respectively: CX3CL1 (1:1,000, ab25088, Abcam) and β-tubulin (1:1,000, Santa Cruz Biotechnology, Heidelberg, Germany) for 2 h at RT. Then NC was washed for 10 min × 3 in TBST and was incubated with secondary antibody peroxidase-conjugated goat anti-rabbit IgG (1:5,000, ZSGB-BIO, Beijing, China) for 1 h at RT. Then NC was washed for 10 min × 3 in TBST and treated with enhanced chemiluminescence reagents (Pierce Biotechnology, Rockford, IL, USA). The blots were exposed to film and analyzed using Quantity One 1-D analysis software (UVP, Upland, CA, USA). The relative protein level was calculated from the ratio of the cytokine absorbance to their corresponding β-tubulin to correct for small differences in protein loading.

### Statistical Analysis

Values are presented as the mean ± the standard error of the mean (SEM). The data were analyzed using One-way ANOVA followed by a Dunnett’s *post hoc* test or Two-way ANOVA followed by a Tukey’s *post hoc* test, where appropriate. The level of significance was set at *p* < 0.05.

## Results

### Effects of FGF2 on Depressive-Like Behaviors Induced by LPS Administration

The effects of FGF2 infusions on behavioral responses induced by LPS administration were shown in Figure 1. In the SPT, a two-way ANOVA identified a significant LPS effect (*F*_(1,20)_ = 12.0, *p* < 0.01) and LPS × FGF2 interaction (*F*_(1,20)_ = 4.8, *p* < 0.05) on saccharin preference (Figure 1B). A *post hoc* analysis showed that FGF2 reversed the decreased saccharin preference in the LPS-treated rats (*p* < 0.05), indicating that the neuroinflammation-induced anhedonia was ameliorated by FGF2 infusions. There were no differences in total liquid intake among groups, indicating that the physical function of drinking was not changed after LPS and FGF2 infusions. In the OFT, a two-way ANOVA identified a significant LPS effect (*F*_(1,20)_ = 15.99, *p* < 0.001) and FGF2 effect (*F*_(1,20)_ = 11.43, *p* < 0.01) on distance traveled. A *post hoc* analysis indicated that FGF2 reversed the decreased distance traveled in the LPS-treated rats (*p* < 0.05, Figure 1C). The rearing times in the OFT was also influenced by FGF2 infusions. A two-way ANOVA identified a significant LPS effect (*F*_(1,20)_ = 15.49, *p* < 0.001) and FGF2 effect (*F*_(1,20)_ = 9.92, *p* < 0.01) on rearing times. A *post hoc* analysis indicated that FGF2 reversed the decreased rearing times in the LPS-treated rats (*p* < 0.05, Figure 1D). These data indicated that the reduced locomotor activity and exploration behavior were reversed by FGF2 infusions in LPS-treated rats.

### Effects of FGF2 on Microglia Activation in Hippocampus

Effects of FGF2 and LPS on microglia activation were shown in Figure 2. We stained the sections of hippocampus with Iba1, the identification marker of microglia (Imai and Kohsaka, 2002; Yirmiya et al., 2015). As shown in Figure 2A, we observed Iba1 labeling in both cell bodies and branches, therefore we compared the percentage of Iba1 positive staining area among groups. A two-way ANOVA indicated a significant LPS effect (*F*_(1,12)_ = 17.70, *p* < 0.01), FGF2 effect (*F*_(1,12)_ = 49.85, *p* < 0.001), and LPS × FGF2 interaction (*F*_(1,12)_ = 5.71, *p* < 0.05) on Iba1 area in hippocampus (Figure 2B). A *post hoc* analysis indicated that Iba1 area was significantly increased in LPS treated rats compared with saline control rats (*p* < 0.01). FGF2 decreased the Iba1 area in LPS treated rats (*p* < 0.001), indicating that FGF2 reversed the LPS-induced microglia activation. We also counted the density of Iba1 positive cells to examine the microglia proliferation. A two-way ANOVA identified a significant LPS effect (*F*_(1,12)_ = 75.38, *p* < 0.001), FGF2 effect (*F*_(1,12)_ = 97.25, *p* < 0.001), and LPS × FGF2 interaction (*F*_(1,12)_ = 54.39, *p* < 0.001) on Iba1 cell density in hippocampus (Figure 2C). A *post hoc* analysis indicated that Iba1 cell density was significantly increased in LPS treated rats compared with saline control rats (*p* < 0.001). FGF2 decreased the Iba1 cell density in LPS treated rats (*p* < 0.001), indicating that the LPS-induced microglia proliferation was alleviated by FGF2 infusions.

The soma area and processes length were assessed to compare the morphology of microglia among groups. Microglial shift from ramified status to activated status was reflected by swollen soma and reduced processes length (Kettenmann et al., 2011). A two-way ANOVA identified a significant LPS effect (*F*_(1,12)_ = 60.24, *p* < 0.001), FGF2 effect (*F*_(1,12)_ = 38.97, *p* < 0.001), and LPS × FGF2 interaction (*F*_(1,12)_ = 45.95, *p* < 0.001) on microglia soma area in hippocampus (Figure 2D). A *post hoc* analysis indicated that soma area was significantly increased in LPS treated rats compared with saline control rats (*p* < 0.001). FGF2 decreased the soma area in LPS treated rats (*p* < 0.001). A two-way ANOVA identified a significant LPS effect (*F*_(1,12)_ = 126, *p* < 0.001), and LPS × FGF2 interaction (*F*_(1,12)_ = 29.63, *p* < 0.001) on microglia processes length in hippocampus (Figure 2E). A *post hoc* analysis indicated that processes length was significantly decreased in LPS treated rats compared with saline control rats (*p* < 0.001). FGF2 increased the processes length in LPS treated rats (*p* < 0.01). These data indicated that the LPS-induced microglial morphology changes were reversed by FGF2 infusions.

### Effects of FGF2 on Cytokines and CX3CL1 Expression in Hippocampus

Activated microglia modulate the neuronal microenvironment by releasing cytokines (Block et al., 2007). To examine the effects of FGF2 on inflammatory state in the hippocampus, the levels of cytokines including IL-1β, IL-6, TNF-α and IL-10 were measured. A two-way ANOVA identified a significant LPS effect (*F*_(1,18)_ = 35.67, *p* < 0.001) and LPS × FGF2 interaction (*F*_(1,18)_ = 4.90, *p* < 0.05) on IL-1β level in hippocampus. A *post hoc* analysis indicated that IL-1β level was significantly increased in LPS treated rats compared with saline control rats (*p* < 0.001). This effect of LPS was insignificant in rats receiving FGF2 infusions (Figure 3A). A two-way ANOVA identified a significant LPS effect (*F*_(1,16)_ = 51.06, *p* < 0.001), FGF2 effect (*F*_(1,16)_ = 5.38, *p* < 0.05), and LPS × FGF2 interaction (*F*_(1,16)_ = 5.07, *p* < 0.05) on IL-6 level in hippocampus. A *post hoc* analysis indicated that IL-6 level was significantly increased in LPS treated rats compared with saline control rats (*p* < 0.001). FGF2 decreased the production of IL-6 in LPS treated rats (*p* < 0.05, Figure 3B). A two-way ANOVA identified a significant LPS effect (*F*_(1,17)_ = 22.09, *p* < 0.001) and LPS × FGF2 interaction (*F*_(1,17)_ = 12.89, *p* < 0.01) on TNF-α level in hippocampus. A *post hoc* analysis indicated that TNF-α level was significantly increased in LPS treated rats compared with saline control rats (*p* < 0.001). This effect of LPS was insignificant in rats receiving FGF2 infusions. FGF2 decreased the production of TNF-α in LPS treated rats (*p* < 0.05, Figure 3C). A two-way ANOVA identified a significant LPS effect (*F*_(1,17)_ = 8.94, *p* < 0.01) on IL-10 level in hippocampus. A *post hoc* analysis indicated that IL-10 level was increased by FGF2 in rats with LPS challenges (*p* < 0.05, Figure 3D). These data indicated that FGF2 alleviated the pro-inflammatory cytokines release induced by LPS, and promoted the anti-inflammatory cytokine release after LPS treatment.

CX3CL1 is mainly expressed by neurons to keep microglia in quiescence (Biber et al., 2007; Corona et al., 2010). The effects of FGF2 on the protein expression of CX3CL1 in hippocampus were further examined by western blot test (Figure 3E). A two-way ANOVA indicated a significant LPS effect (*F*_(1,20)_ = 16.17, *p* < 0.001), FGF2 effect (*F*_(1,20)_ = 13.90, *p* < 0.01) on the CX3CL1 expression in hippocampus (Figure 3F). A *post hoc* analysis indicated that CX3CL1 expression was significantly inhibited in LPS treated rats compared with saline control rats (*p* < 0.01). FGF2 significantly increased the CX3CL1 expression in LPS treated rats (*p* < 0.01). These data indicated that the process of FGF2 regulating microglia activation involves the CX3CL1 signal.

### The Behavioral Effects of SU5402

To examine the behavioral responses after blocking FGF2 signaling, two doses of FGFR inhibitor (SU5402) were used in the Experiment 3. The behavioral effects of SU5402 were shown in Figure 4. In SPT, a one-way ANOVA identified a significant treatment effect (*F*_(1,15)_ = 5.79, *p* < 0.05) on saccharin preference. A *post hoc* analysis indicated that saccharin preference was decreased by 5 μg dose of SU5402 (*p* < 0.01, Figure 4B). Whereas the total liquid intake in SPT did not differ among groups, indicating that SU5402 induced anhedonia but not influenced physical function of drinking. In OFT, one-way ANOVA identified significant treatment effects on distance traveled (*F*_(1,15)_ = 7.10, *p* < 0.01). *Post hoc* analysis showed that 5 μg dose of SU5402 decreased the distance traveled (*p* < 0.01, Figure 4C) and rearing times (*p* < 0.05, Figure 4D), indicating that SU5402 infusions induced reduction in locomotor activity and exploration in rats.

### Effects of SU5402 on Microglia Activation in Hippocampus

**Figure 5:**
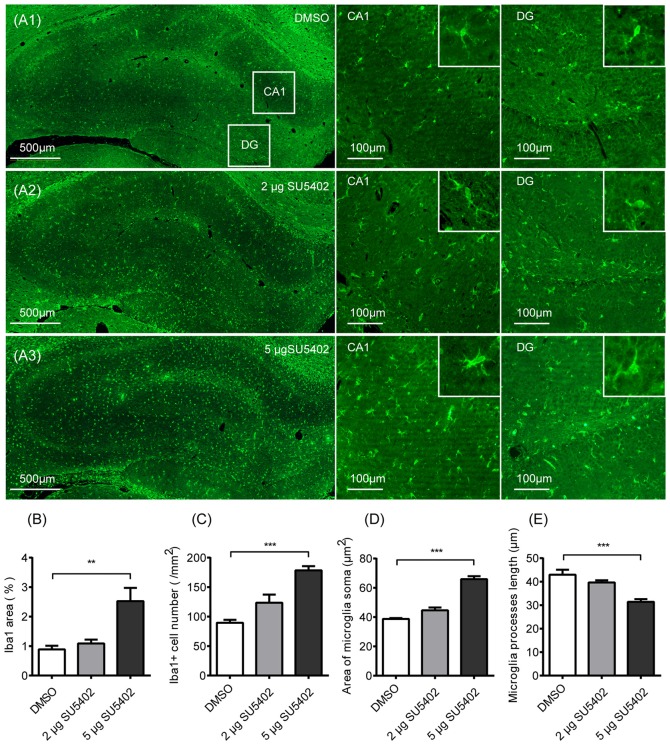
Impact of FGFR inhibitor (SU5402) on the microglia activation in hippocampus. **(A1–A3)** Representative photographs of Iba1 expression in hippocampus. Compared with the DMSO group, there were increased expression of microglia identification marker Iba1 and more Iba1 positive cells in CA1 and DG with the swollen soma and retracted processes in 5 μg dose of SU5402 group. **(B)** The percentage of Iba1 positive area was increased by 5 μg dose of SU5402. **(C)** The density of Iba1 positive cells was increased by 5 μg dose of SU5402. **(D)** The area of microglia soma was increased by 5 μg dose of SU5402. **(E)** The length of microglial processes was decreased by 5 μg dose of SU5402. One-way ANOVA with Dunnett’s multiple comparisons test, *n* = 4 per group. Data are presented as the means ± SEM. **p < 0.01, ***p < 0.001. DMSO, dimethyl sulfoxide; DG, dentate gyrus.

Effects of FGFR inhibitor (SU5402) on the microglia activation were shown in Figure 5. As shown in Figure 5A, the expression of microglia identification marker Iba1 were increased after SU5402 infusions. A one-way ANOVA identified a significant treatment effect (*F*_(2,9)_ = 10.20, *p* < 0.05) on Iba1 area in hippocampus. A *post hoc* analysis indicated that the Iba1 area was increased by 5 μg dose of SU5402 (*p* < 0.01, Figure 5B). A one-way ANOVA identified a significant treatment effect (*F*_(2,9)_ = 22.50, *p* < 0.001) on Iba1 cell density in hippocampus. A *post hoc* analysis indicated that the Iba1 cell density was increased by 5 μg dose of SU5402 (*p* < 0.001, Figure 5C). A one-way ANOVA identified a significant treatment effect (*F*_(2,9)_ = 73.84, *p* < 0.001) on microglia soma area in hippocampus. A *post hoc* analysis indicated that the microglia soma area was increased by 5 μg dose of SU5402 (*p* < 0.001, Figure 5D). A one-way ANOVA identified a significant treatment effect (*F*_(2,9)_ = 15.83, *p* < 0.01) on microglia processes length in hippocampus. A *post hoc* analysis indicated that the processes length was decreased by 5 μg dose of SU5402 (*p* < 0.001, Figure 5E). These data indicated that, at a concentration of 5 μg, SU5402 evoked microglia activation, increased microglia proliferation and shifted more microglia into activated morphology.

### Effects of SU5402 on Cytokines and CX3CL1 Expression in Hippocampus

**Figure 6:**
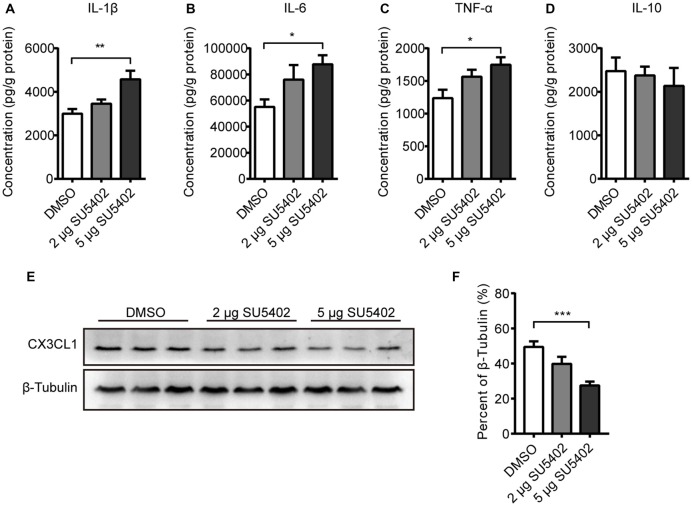
Impact of FGFR inhibitor (SU5402) on cytokines and CX3CL1 expression in hippocampus. **(A–C)** The IL-1β, IL-6 and TNF-α levels were increased by 5 μg dose of SU5402. **(D)** SU5402 did not influenced the IL-10 level. **(E)** Representative western blots of CX3CL1 and corresponding normalized control β-Tubulin in hippocampus. **(F)** The expression of CX3CL1 was decreased by 5 μg dose of SU5402 infusions. One-way ANOVA with Dunnett’s multiple comparisons test, *n* = 5 or 6 per group. Data are presented as the means ± SEM. *p < 0.05, **p < 0.01; ***p < 0.001. DMSO, dimethyl sulfoxide.

Effects of FGFR inhibitor (SU5402) on cytokine level in hippocampus were shown in Figures 6A–D. A one-way ANOVA identified a significant treatment effect (*F*_(2,13)_ = 7.52, *p* < 0.01) on IL-1β level. A *post hoc* analysis indicated that the IL-1β level was increased by 5 μg dose of SU5402 (*p* < 0.01, Figure 6A). A one-way ANOVA identified a significant treatment effect (*F*_(2,13)_ = 4.17, *p* < 0.05) on IL-6 level in hippocampus. A *post hoc* analysis indicated that the IL-6 level was increased by 5 μg dose of SU5402 (*p* < 0.05, Figure 6B). A one-way ANOVA identified a significant treatment effect (*F*_(2,13)_ = 4.82, *p* < 0.05) on TNF-α level in hippocampus. A *post hoc* analysis indicated that the TNF-α level was increased by 5 μg dose of SU5402 (*p* < 0.05, Figure 6C). The IL-10 level was not influenced by SU5402 in hippocampus (Figure 6D). The effects of SU5402 on the protein expression of CX3CL1 in hippocampus were examined by western blot test (Figure 6E). A one-way ANOVA identified a significant treatment effect (*F*_(1,15)_ = 11.84, *p* < 0.001) on CX3CL1 expression in hippocampus. A *post hoc* analysis indicated that the CX3CL1 expression was inhibited by 5 μg dose of SU5402 (*p* < 0.001, Figure 6F). These data indicated that, at a concentration of 5 μg, SU5402 infusions increased the production of pro-inflammatory cytokines and decreased the expression of CX3CL1 in hippocampus.

## Discussion

The present study demonstrated that central LPS challenges induced depressive-like behaviors and inflammatory activation of microglia in hippocampus, manifested as the increased expression of microglia identification marker Iba1, microglial proliferation, swollen microglial soma, and retraction of microglial processes. FGF2 infusions significantly reversed depressive-like behaviors, decreased the microglial proliferation and inhibited microglial morphological changes induced by LPS. While FGF2 ameliorated the increased production of pro-inflammatory cytokines including IL-1β, IL-6 and TNF-α, it enhanced the level of IL-10, an anti-inflammatory cytokine in LPS-treated rats. The expression of CX3CL1 was also inhibited by LPS treatment and reversed by FGF2 infusions in the present study. Vice versa, blocking FGFR using SU5402 caused microglia activation in hippocampus, increased release of pro-inflammatory cytokines, decreased the CX3CL1 expression, and evoked the depressive-like behaviors. Two lines of evidence demonstrate that FGF2 modulate microglia activation in hippocampus which may underlie the pathogenesis of neuroinflammation-associated depression.

The neuroprotective effects of FGF2 have been demonstrated in many studies that FGF2 promotes proliferation of neural progenitor cells in neurogenic niches, enhances synaptic plasticity and axonal branching and ameliorates the behavioral deficiency in neurodegenerative diseases (Baron et al., 2012; Elsayed et al., 2012; Kiyota et al., 2012; Woodbury and Ikezu, 2014; Tang et al., 2017). Our previous study also demonstrated that FGF2 reversed depressive-like behaviors and the impaired hippocampal neurogenesis induced by neuroinflammation (Tang et al., 2017). In the present study, we further found that the activation of microglia induced by LPS was reversed by FGF2 infusions, which provided new information for understanding the pharmacologic mechanism of antidepressant action of FGF2. Because the activation of microglia, the primary resident immune cell in the brain, is the core of neuroinflammatory state, some forms of depression have been suggested as a microglial disease (Kettenmann et al., 2011; Yirmiya et al., 2015; Singhal and Baune, 2017). However, there is no direct evidence to show whether the antidepressant role of FGF2 in neuroinflammation-associated depression involves the actions on microglia in the brain, the present finding is the first answer to this issue.

Depending on the integration of regulatory signals, activation of microglia could be viewed as a linear spectrum, on which M1 represents one extreme and M2 represents the other (Mosser and Edwards, 2008). M1 microglia produce inflammatory cytokines and reactive oxygen species and generate a detrimental microenvironment for neurons, while M2 microglia secrete neurotrophic factors and anti-inflammatory mediators and provide a supporting microenvironment for neurons (Kettenmann et al., 2011). In the present study, LPS resulted in M1 microglia indexed by high levels of the pro-inflammatory cytokines. FGF2 infusions ameliorated the LPS-induced production of microglial M1 markers, and increased the M2 marker indexed by IL-10, suggesting a shift from M1 to M2 phenotype. The neurotoxic effects of pro-inflammatory cytokines have been demonstrated in many studies that these cytokines account for the pathogenesis of depressive disorder and have been considered as the basis of inflammatory hypothesis of depression (Maes et al., 2011; Dantzer, 2012). Indeed, in our previous data, significant release of pro-inflammatory cytokines was observed in both chronic swim stress-induced and central LPS challenge-induced models of depression (Guan et al., 2015; Tang et al., 2016).

Interestingly, the present data showed that FGF2 not only ameliorated the increased production of pro-inflammatory cytokines in the neuroinflammation induced model of depression, but also enhanced the level of IL-10, a representative anti-inflammatory cytokine. IL-10 has been reported to exert key functions to maintain the balance between pro- and anti-inflammatory and prevent inflammatory pathologies (Ouyang et al., 2011). Recent evidence demonstrates that IL-10 could act as a growth factor on the proliferation of neural progenitor cell and upregulate neurogenesis in adult brain (Perez-Asensio et al., 2013; Pereira et al., 2015). Thus, the neuroprotective properties of IL-10, the recovery of pro- and anti-inflammatory cytokines balance, and the transition of microglial phenotypes may all contribute to the antidepressant effects of FGF2.

As to the function of microglia and neurons, it should be noted that, on one hand, enhanced microglia activation and amplified pro-inflammatory cytokine production can impair normal neuronal function and, on the other hand, neurons not only passively accept the regulation of microglia but also actively control microglia (Tian et al., 2009, 2012). Among the candidate pathways which could control microglial response, CX3CL1/CX3CR1 signaling provides insights into microglia-neuron interactions (Sheridan and Murphy, 2013; Limatola and Ransohoff, 2014). Damaged neurons activate microglia through CX3CL1/CX3CR1 signaling to help clear debris, release cytokines and repair tissue (Liu et al., 2015; Dorfman et al., 2017). When the inflammation is excessive, neurons inhibit the microglia activation through CX3CL1/CX3CR1 signal pathway (Zanier et al., 2016). The present data showed that FGF2 reversed the decline of CX3CL1 protein expression induced by LPS in hippocampus, suggesting that CX3CL1 signaling, which deliver a calming control to microglia, participates in the effects of FGF2 on microglia. Our results are in coincidence with the report that microglia activation is amplified and prolonged in the aged brain which shows reduced expression level of CX3CL1 within the hippocampus compared to adults, and restoring CX3CL1 level increases new cells in the granule cell layer (Bachstetter et al., 2011; Norden and Godbout, 2013). Based on our finding of CX3CL1 and others, further study should assess the interactions between CX3CL1 on neurons and CX3CR1 on microglia to reveal communication mechanisms of microglial regulation in neuroinflammation-associated depression.

To further confirm the changes of involved elements resulted from FGF2 infusions, the effects of FGFR inhibitor, SU5402, on behavioral, cellular and molecular responses were investigated. We examined the effects of central SU5402 infusions at two concentrations. According to previous studies, the dose of 2 μg could effectively inhibit cell proliferation and downregulate the cascaded signals such as protein kinase C (PKC) and extracellular signal-regulated kinase (ERK; Fujita-Hamabe et al., 2011; Kalluri et al., 2013), and cause, at approximate this concentration, significant reductions of adult neurogenesis in hippocampus and prefrontal cortex (Mudò et al., 2007; Elsayed et al., 2012). However, in the present study, the dose of 2 μg did not result in depressive-like behavior and microglia activation in hippocampus. This result could be due to that the dosage of 2 μg is too low to work because it was once only used in mice rather than rats. Then we increased the dose to 5 μg, and observed significant depressive-like behaviors and microglia activation in hippocampus, which is the first evidence showing that blocking FGFR signaling induces depressive-like behaviors in rats. Our findings that inhibition of FGFR signaling resulted in microglia activation in hippocampus is consistent with previous finding that FGF2^−/–^ mice display increased infiltration of T cells and microglia in the CNS (Rottlaender et al., 2011). In the present study, the infusions of SU5402 at the dose of 5 μg increased the production of pro-inflammatory cytokines including IL-1β, IL-6 and TNF-α but did not change the level of anti-inflammatory IL-10, suggesting microglia were shifted to M1 phenotype. In line with this finding, the dose of 5 μg SU5402 downregulated the protein expression of CX3CL1, indicating neuroprotective action of microglia was also inhibited by SU5402. However, in the present study, the effect of SU5402 on depressive-like behavior in normal rats is not sufficient to determine the role of FGFR in the inflammation-induced depression model. The dosage and efficiency of SU5402, as an inhibitor of FGF2, also needs more examination. One of considerations is that SU5402 may not be selective for blocking FGFR and may also affect other growth factor receptors including vascular endothelial growth factor receptor and platelet-derived growth factor receptor (Boschelli, 1999; Grand et al., 2004; Liang et al., 2012). Further studies are needed to investigate whether and how FGF2 inhibitor SU5402 can prevent the antidepressant effect of FGF2 and provide more experimental evidence for the relevance of FGF2/FGFR pathway in inflammation-induced depression.

In summary, the present results demonstrate that the antidepressant effects of FGF2 and the depressive effects of FGFR blocker involve mediation of microglia activation in hippocampus. Enhanced FGF2 signaling inhibits microglia activation and hippocampal pro-inflammatory cytokines release induced by LPS, reverses the decline of CX3CL1 protein expression, and ameliorates depressive-like behaviors. In contrast, blocking FGFR signaling increases microglia activation, results in more release of pro-inflammatory cytokines, decreases CX3CL1, and evokes depressive-like behaviors. These findings suggest that FGF2/FGFR signaling pathway may play a crucial role in maintaining microglial resting status and the balance of pro- and anti-inflammatory cytokines, which provide new insights into the understanding of the antidepressant effect of FGF2 as well as the mechanism of neuroinflammation-associated depression.

## Author Contributions

W-jL supervised the overall research. M-mT performed the experiments and collected the data. Y-qP, Y-cL and M-mT prepared materials and interpreted the data. M-mT and W-jL wrote and revised the manuscript.

## Conflict of Interest Statement

The authors declare that the research was conducted in the absence of any commercial or financial relationships that could be construed as a potential conflict of interest.
